# DNA Topoisomerase II Modulates Insulator Function in *Drosophila*

**DOI:** 10.1371/journal.pone.0016562

**Published:** 2011-01-27

**Authors:** Edward Ramos, Eduardo A. Torre, Ashley M. Bushey, B. V. Gurudatta, Victor G. Corces

**Affiliations:** 1 Department of Biology, Emory University, Atlanta, Georgia, United States of America; 2 Department of Biology, Johns Hopkins University, Baltimore, Maryland, United States of America; Texas A&M University, United States of America

## Abstract

Insulators are DNA sequences thought to be important for the establishment and maintenance of cell-type specific nuclear architecture. In *Drosophila* there are several classes of insulators that appear to have unique roles in gene expression. The mechanisms involved in determining and regulating the specific roles of these insulator classes are not understood. Here we report that DNA Topoisomerase II modulates the activity of the Su(Hw) insulator. Downregulation of Topo II by RNAi or mutations in the *Top2* gene result in disruption of Su(Hw) insulator function. This effect is mediated by the Mod(mdg4)2.2 protein, which is a unique component of the Su(Hw) insulator complex. Co-immunoprecipitation and yeast two-hybrid experiments show that Topo II and Mod(mdg4)2.2 proteins directly interact. In addition, mutations in *Top2* cause a slight decrease of Mod(mdg4)2.2 transcript but have a dramatic effect on Mod(mdg4)2.2 protein levels. In the presence of proteasome inhibitors, normal levels of Mod(mdg4)2.2 protein and its binding to polytene chromosomes are restored. Thus, Topo II is required to prevent Mod(mdg4)2.2 degradation and, consequently, to stabilize Su(Hw) insulator-mediated chromatin organization.

## Introduction

Eukaryotes use complex mechanisms to regulate spatial and temporal patterns of gene expression. At the chromatin level, much research has focused on the role of histone modifications and chromatin remodeling complexes in promoting or preventing transcription [1], [2]. In addition, the highest order of chromatin structure, the chromosome, also participates in the establishment and maintenance of gene expression patterns [3], [4], [5]. Organization of chromatin at this level is complex, requiring an intricate balance between DNA compaction and accessibility to the transcription and replication machineries. A wealth of information accumulated during the last few years implicates chromatin insulators in the establishment of higher-order chromatin structure through the formation of chromatin loops and subsequent regulation of gene expression [6].

Enhancer-blocking insulators are DNA sequences defined by their ability to interfere with enhancer-promoter communication whereas barrier insulators have the ability to shield transgenes from position effects caused by surrounding chromatin [7], [8]. The properties of enhancer blocking insulators can be explained by their role in mediating inter- and intra-chromosomal interactions that result in the establishment and/or maintenance of chromatin loops [9], [10], [11], [12], [13]. The formation of these loops can result in multiple insulators from distinct genomic loci coalescing via protein-protein interactions to form multi-complex entities termed insulator bodies. These insulator bodies may form functional chromatin domains isolating sequences within different loops and preventing interference from regulatory regions in one loop on genes located in other loops [14], [15]. Recently, the nature of insulator bodies as entities formed by multiple insulator sites coalescing at a specific nuclear location has been brought into question based on the identification of a mutation in *mod(mdg4)* that affects insulator function without visibly disrupting the integrity of insulator bodies [16]. As an alternative, these authors suggest that insulator bodies may be protein aggregates. Nevertheless, the results can be also explained if the *mod(mdg4)* allele is a hypomorph that affects the insulator activity of the gypsy retrotransposon insulator, which has 12 copies of the Su(Hw) binding site, but not the function of endogenous insulators present in the *Drosophila* genome, which only contain 1–2 copies of this sequence.

Evidence from genome-wide association studies suggests that insulators may create a cell-type specific nuclear architecture that is important for the establishment and/or maintenance of linage specific gene expression and genome organization [17], [18], [19], [20], [21]. Recent results suggest that three different *Drosophila* insulators utilize different DNA binding proteins to recognize different sites in the genome but share CP190, which is the main component responsible for inter-insulator interactions [17]. These studies indicate that these three insulator subclasses may serve unique functions in the cell. As a consequence, cells may have mechanisms to independently regulate the function of each insulator subclass. However, the mechanisms underlying the regulation of the activity of each of these insulators are not well understood.

The main *Drosophila* insulators characterized to date are defined by their DNA binding proteins, Su(Hw), dCTCF and BEAF [13]. The Su(Hw) insulator has two other core protein components, Mod(mdg4)2.2 and CP190 [15], [22], [23]; in addition, this insulator complex contains RNA as well as other proteins that may serve a regulatory function [24], [25]. Here we present evidence suggesting that DNA Topoisomerase II (Topo II) is also required to modulate the activity of the Su(Hw) insulator. Topo II has been shown to be important for critical cellular functions such as transcription, replication, recombination and genome stability in addition to its function in the organization of chromatin architecture [26], [27], [28]. Mechanistically, Topo II functions to alter DNA topology by catalyzing the ATP-dependent passage of one DNA double helix through another by breaking and religating one of the DNA strands while transporting the second [26], [29], [30], [31]. Empirical studies have demonstrated that Topo II may function to relieve supercoiling attributed to RNA polymerase-driven transcription [32], [33]. Data presented here suggest that Topo II is also needed for proper Su(Hw) insulator function in *Drosophila*. Loss of Topo II leads to a reversion of *gypsy*-induced phenotypes along with the inability of Su(Hw)/Mod(mdg4)2.2 to form insulator bodies. This effect is specific for the Su(Hw) insulator subclass and does not affect insulator bodies involving other insulator proteins. The effect of Topo II on Su(Hw) insulators appears to be mediated by the Mod(mdg4)2.2 protein; decrease of Topo II function leads to the degradation of Mod(mdg4)2.2 through a proteasome-dependent pathway. These results suggest a novel mechanism used to regulate a specific subset of insulator-mediated chromatin organization.

## Results

### Reduction of Topo II via RNAi affects *gypsy*-induced phenotypes

**Figure 1:**
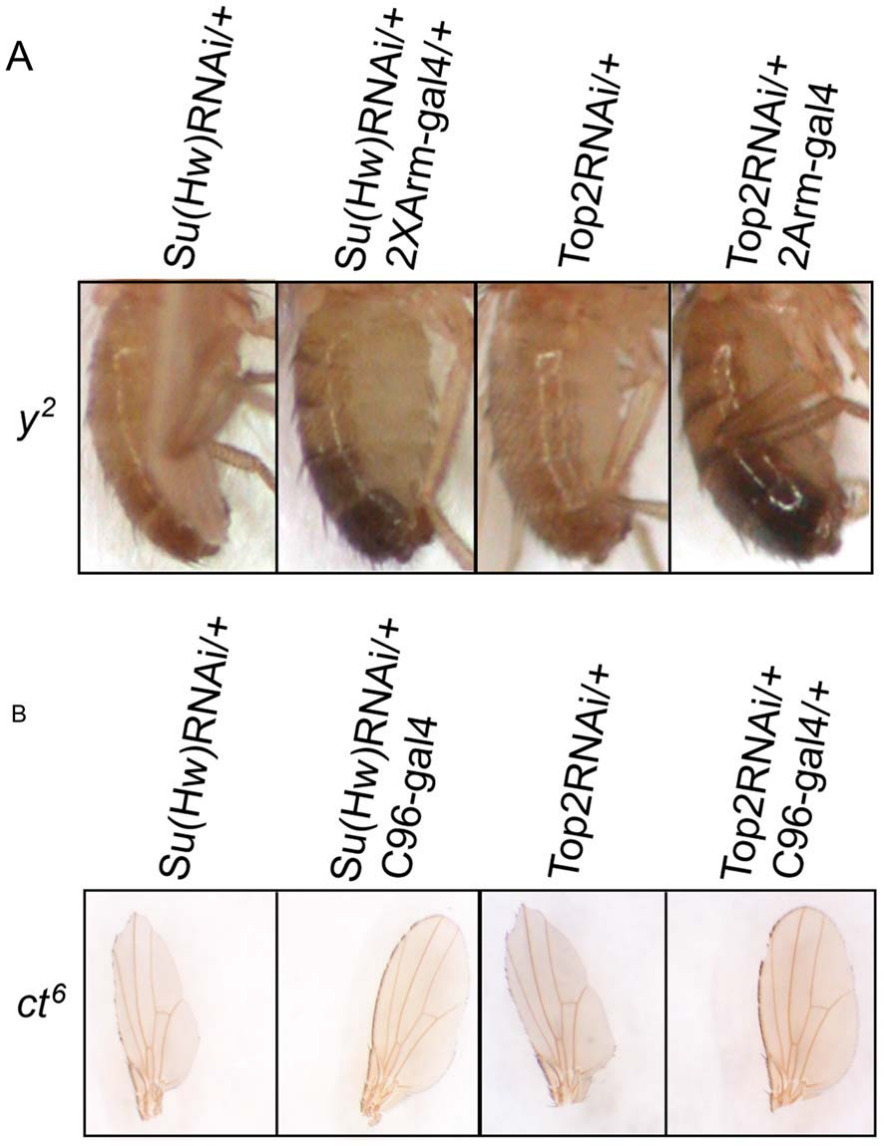
*Top2* RNAi knockdown affects *gypsy* phenotypes. (A) Transgenic *UAS-Su(Hw)RNAi* and *UAS-Top2RNAi* lines were used to knockdown Su(Hw) and Topo II in a *y^2^ct^6^* mutant background. The transgenic RNAi lines alone do not affect the *y^2^* phenotype. In the control, Su(Hw) was knocked down using 2 copies of the *Arm-Gal4*driver, causing a reversion in coloration of the abdomen to black and suggesting that the *gypsy* insulator is no longer functional. Reduction of *Top2* also results in decreased function of the *gypsy* insulator, changing the coloration of the abdomen from yellow to black. (B) The same *UAS-Su(Hw)RNAi* and *UAS-Top2RNAi* lines were used to test the effect of loss of Su(Hw) and Topo II on the *gypsy* induced *cut* phenotype. The RNAi lines alone have no effect on the *ct^6^* phenotype. However, reduction of Su(Hw) or Topo II using the wing driver *C96-Gal4* cause a reversion from a cut to a more wild type wing.

To test the role of Topo II in insulator function we used RNAi to decrease the amount of Topo II (*Top2*) gene expression in flies. This allowed us to examine the effect of downregulation of this protein on the phenotype of *y^2^wct^6^* flies carrying the *gypsy* retrotransposon inserted into the *yellow* (*y*) and *cut* (*ct*) genes. The *gypsy* insertion affects the communication between upstream enhancers and downstream promoters, causing yellow body and cut wing phenotypes when insulator proteins bind to the *gypsy* insulator sequence [15] (Figure 1). In a *y^2^wct^6^* background, a transgenic fly expressing a *UAS-Top2RNAi* construct was used with various *Gal4* drivers to reduce Topo II expression in different tissues. The *UAS-Top2RNAi* transgenic fly specifically targets the*Top2* gene and has no off target sites [34]. First, to test effects on the *y^2^* phenotype, the weak but ubiquitously expressed driver *Arm-Gal4* [35] was used to knockdown *Top2* expression. Quantitative real-time PCR (qRT-PCR) analysis shows that the transcript levels of *Top2* are reduced in the *Arm-Gal4;UAS-Top2RNAi* flies (Figure S1A). As a positive control for the effects on the *y^2^* phenotype, we used the *Arm-Gal4* line in combination with a *UAS-Su(Hw)RNAi* line, which results in a decrease in *gypsy* insulator function manifested by a change in body color from yellow to black (Figure 1A). Loss of Su(Hw) provides an example of the phenotype expected by a reversion of the insulation caused by the *gypsy* insertion [36]. Similar results are seen when the *Arm-Gal4* driver is crossed to the *UAS-Top2RNAi* line, with a reversion from a yellow body to a black body phenotype (Figure 1A). This suggests that loss of Topo II has an effect on the function of the *gypsy* insulator similar to that of Su(Hw). To confirm this finding, we further tested whether loss of Topo II also affects the *cut* wing phenotype in the *ct^6^* allele by crossing the *UAS-Top2RNAi* and *UAS-Su(Hw)RNAi* lines to a *C96-Gal4* driver. *C96-Gal4* is expressed in the prospective wing margin region of the developing wing disc where the *ct* gene is expressed and, in combination with the *UAS-Top2RNAi* line, reduces the level of Topo II in the wing imaginal disc cells that give rise to the dorsal-ventral margin (Figure S1B). When the *C96-Gal4* driver is crossed with the *UAS-Su(Hw)RNAi* control, the *ct* phenotype reverts to wild type (Figure 1B). A similar change in phenotype is observed when the *C96-Gal4* driver is combined with the *UAS-Top2RNAi* line (Figure 1B), again suggesting that Topo II interacts with *gypsy* insulator components to regulate insulator function.

### Loss of Topo II in *Drosophila* diploid cells affects insulator body formation

In *Drosophila* the insulator proteins Su(Hw), Mod(mdg4)2.2, dCTCF, and CP190 co-localize to punctate foci within the nucleus termed insulator bodies. These insulator bodies are thought to result from several insulators from different genomic locations coming together and looping the intervening chromatin fiber [14], [15], [37], [38], [39]. Since insulator function correlates with the formation of insulator bodies, and downregulation of Topo II affects the activity of the *gypsy* insulator, we examined whether loss of Topo II has an effect on insulator body formation using RNAi to knockdown this protein in *Drosophila* cultured cells [40]. Using amplicons targeting the second and fourth exons of *Top2*, double stranded RNAs (dsRNAs) were made and used for the knockdown assays. A schematic representation of the *Top2* locus indicating the location of the amplicons is shown in Figure S2A. Both the second and fourth exon amplicons have no off target sites and only target transcripts made by the *Top2* gene. As a control for these experiments, a *lacZ* amplicon was used to make dsRNAs of *lacZ* to initiate the RNAi machinery and rule out any RNAi pathway specific effects. Similar to previous data by the Carmena group [41], we find that dsRNAs targeting *Drosophila Top2* greatly reduce Topo II levels by 72 hours (Figure S2B) and cells can survive without this protein for longer than 6 days.

**Figure 2:**
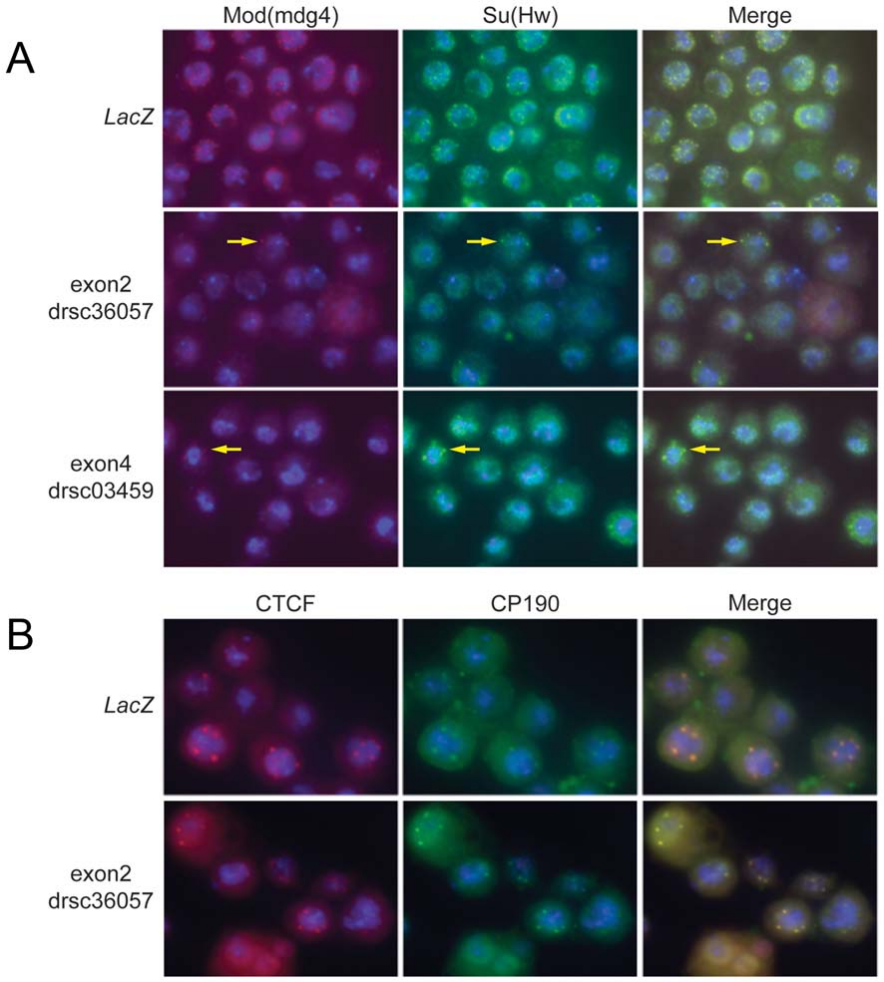
*Top2* RNAi knockdown in diploid cells. dsRNA knockdown of *Top2* using either amplicon targeting exon 2 or exon 4 causes a loss of Mod(mdg4)2.2 (red) and Su(Hw) (green) insulator bodies. (A) In the control, knockdown of *LacZ* has no effect on insulator body formation as indicated by the yellow color in the merge panel. The yellow arrows point to cells, in the knockdown cell population, where the Su(Hw) and Mod(mdg4)2.2 insulator bodies still form. (B) dsRNA knockdown of *Top2* has no effect on dCTCF (red) or CP190 (green) insulator bodies as indicated by the formation of these structures in the *LacZ* control and *Top2* knockdown.

To examine the effect of Topo II knockdown on insulator body formation, *Drosophila* S2 cells were fixed 3–4 days after treatment with dsRNAs and then immunostained with a combination of antibodies against control and insulator proteins. Downregulation of *Top2* disrupts the formation of Mod(mdg4)2.2 and Su(Hw) insulator bodies whereas the distribution of these two proteins is not affected in the control *lacZ* knockdown experiment (Figure 2A). However, normal insulator bodies form in a few cells (yellow arrows) where presumably the knockdown of *Top2* is incomplete. Interestingly, loss of Topo II seems to have no effect on the distribution of CP190 or dCTCF insulator bodies (Figure 2B). Thus, the loss of Topo II negatively affects the ability of only Su(Hw) and Mod(mdg4)2.2 to form insulator bodies. Together these results support findings suggesting that Su(Hw) and dCTCF are part of distinct subfamilies of insulators that are independent of each other but can colocalize in the cell nucleus [13], [37], [42]. In addition, these results suggest that the effects of Topo II on insulator function extend beyond those observed for the *gypsy* retrotransposon and apply to the endogenous Su(Hw) sites that are likely present at insulator bodies.

### Characterization of *Drosophila Top2* mutants

To further elucidate the role of Topo II in insulator function we examined the effect of mutations in the *Top2* gene. We obtained five transgenic lines with P-element insertions in the *Top2* locus from the Bloomington *Drosophila* Stock Center at Indiana University, none of which had been characterized previously. These transgenic strains are *Top2^c05388^* (*Top2^c^*), *Top2^d05357^* (*Top2^d^*), *Top2^f05145^* (*Top2^f^*), *Top2^LA00892^* (*Top2^LA^*) and *Top2^MB04073^* (*Top2^MB^*). Each of these transgenic lines has a P-element inserted in the first intron of the *Top2* gene (Figure S2A). To determine if any of these P-element insertions causes a mutant phenotype, each of the *Top2* alleles was crossed with a *CyO, Act-GFP* balancer line and homozygous *Top2* progeny were identified by the lack of GFP expression beginning at embryogenesis. Using this approach we determined that lines *Top2^c^* and *Top2^f^* are homozygous lethal while the other *Top2* strains are not. qRT-PCR was then used to characterize these *Top2* alleles. *Top2^c^* and *Top2^f^* have no detectable *Top2* transcript while the amount of the *Top2* RNA in the other alleles varies from approximately 40% to 90% of wild type levels (Figure S3A). Western analysis indicates that Topo II levels are only slightly affected in homozygous *Top2^d^*, *Top2^LA^* and *Top2^MB^* individuals while no Topo II protein is detectable in *Top2^c^* and *Top2^f^* mutant alleles (Figure S3).

*Top2^c^* displays the strongest lethal effect when homozygous, with most flies dying as first instar larvae and very few (<5%) escaping to second instar. The weaker *Top2^f^* allele, on the other hand, has its highest mortality rate during second instar with fewer than 5% individuals escaping into the third instar larval stage. To further confirm that the lethality seen with homozygous *Top2^c^* and *Top2^f^* is due to the loss of Topo II function, we placed *Top2^c^* and *Top2^f^* over the deficiency allele *Df(2L)Exel9043* that has a complete deletion of the *Top2* gene and displays lethality during the first instar larva stage. In this combination, both *Top2^c^* and *Top2^f^* die as first instar larvae, similar to what is seen with homozygous *Top2^c^* mutants and *Actin5C-Gal4/UAS-Top2RNAi* animals. Transheterozygous combinations of the *Top2^c^* and *Top2^f^* alleles also show lethality during first instar larvae. Homozygous *Top2^c^* and *Top2^f^* mutants progresses normally through embryogenesis, presumably due to maternal contribution of Topo II, but once hatched, *Top2^c^* and *Top2^f^* animals show a 2–3-day delay in development when compared to wild type larvae reared under identical conditions. The *Top2^f^* third instar escapers are smaller than wild type larvae and have less developed salivary glands and central nervous system while having extremely reduced to completely absent imaginal tissue. A summary of this information is shown in Table 1. Based on the fact that *Top2^c^* and *Top2^f^* are homozygous lethal, do not complement the *Top2* deficiency allele and have undetectable transcript and protein levels, we conclude that these mutations affect the *Top2* gene and could thus be used to study the role of Topo II in insulator protein function.

**Table 1:** Genetic analysis of P-element crosses.

Alleles	*Top2^c^*	*Top2^f^*	*Top2^d^*	*Top2^LA^*	*Top2^MB^*	*Df(2L)*
***Top2^c^***	2^nd^ instar*	2^nd^ instar*	Viable	Viable	Viable	2^nd^ instar*
***Top2^f^***		3^rd^ instar*	Viable	Viable	Viable	2^nd^ instar *
***Top2^d^***			Viable	Viable	Viable	Viable
***Top2^LA^***				Viable	Viable	Viable
***Top2^MB^***					Viable	Viable
***Df(2L)***						2^nd^ instar*

*Homozygous or trans-heterozygous lethal with some 2^nd^ or 3^rd^ instar escapers.

Mutant alleles were crossed in all combinations to determine complementation. The term “viable” indicates that flies have no visible phenotypes, survive to adults and are fertile.

### Mod(mdg4)2.2 is not present at insulator sites in *Top2* mutants

**Figure 3:**
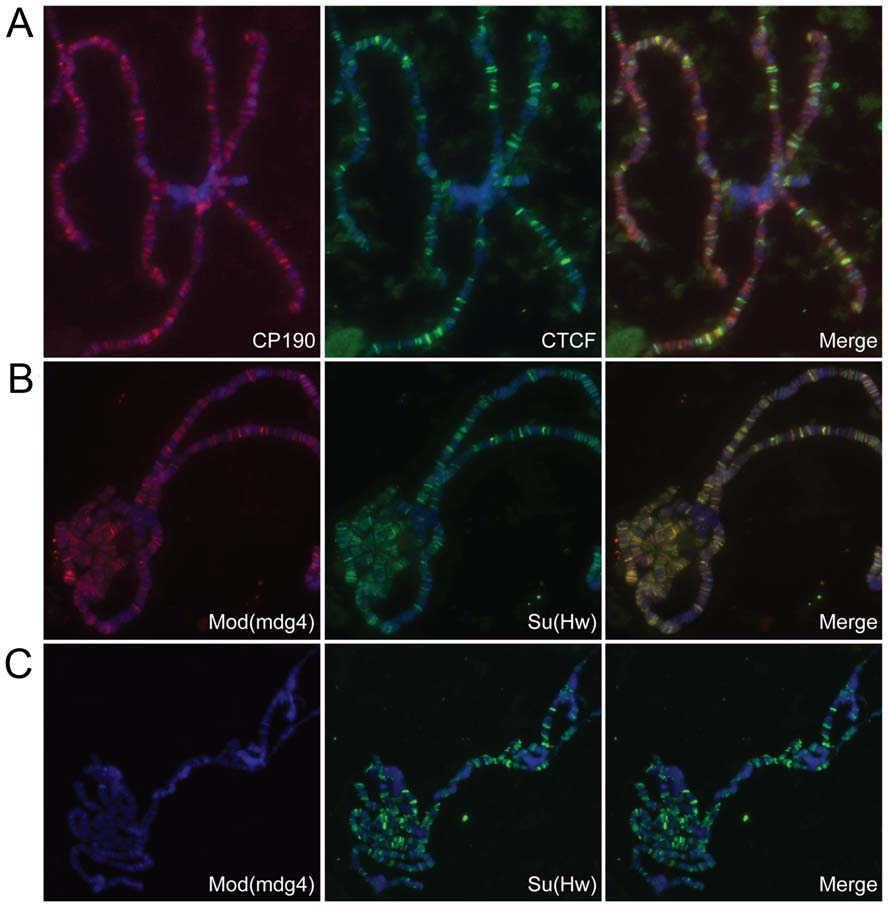
Insulator protein localization on polytene chromosomes from *Top2* mutants. (A) CP190 (red) and dCTCF (green) localize normally to polytene chromosomes from *Top2^f^* mutant larvae. Overlap of CP190 and dCTCF is indicated by the yellow coloration in the merge panel. (B) Mod(mdg4)2.2 (red) and Su(Hw) (green) localization on wild type polytene chromosomes; Mod(mdg4)2.2 and Su(Hw) co-localize extensively as indicated by the overlap in the merge panel (yellow). (C) Only Su(Hw) is present (green) whereas Mod(mdg4)2.2 is absent (no red) in T*op2^f^* mutant polytene chromosomes. In all the panels DAPI is blue.

To address how loss of Topo II may affect insulator function, polytene chromosomes of mutant *Top2^f^* larvae were used to examine the presence of insulator proteins on polytene chromosomes using immunofluorescence microscopy. Since loss of *Top2* via RNAi in S2 cells affects the formation of Su(Hw) and Mod(mdg4)2.2 insulator bodies we wanted to determine whether loss of Topo II in*Top2* mutants also has an effect on the binding of insulator proteins to chromosomes. Polytene chromosomes from *Top2^f^* third instar larvae escapers were first immunostained with antibodies to Topo II; this protein is not present at detectable levels on the polytene chromosomes (Figure S4A). In addition, similar to the results seen with the *Top2* dsRNA knockdown in S2 cells, localization of CP190 and dCTCF on polytene chromosomes is unaffected by the loss of Topo II (Figure 3A). However, unlike the S2 cell knockdown, results indicating that loss of *Top2* inhibits the formation of Su(Hw) insulator bodies, Su(Hw) binding is not affected on polytene chromosomes from*Top2* mutant larvae (Figure 3B–C). This suggests that the inability of Su(Hw) to form insulator bodies is not due to the failure of Su(Hw) to directly bind DNA but may be an effect on the integrity of the overall Su(Hw) complex. To test this possibility, we examined the distribution of Mod(mdg4)2.2 on polytene chromosomes of *Top2^f^* mutant larvae and found that loss of Topo II drastically reduced the levels of Mod(mdg4)2.2 present in polytene chromosomes (Figure 3C and Figure S4B). In addition, Mod(mdg4)2.2 is also absent in diploid cells of the *Top2^f^* mutant, whereas CP190 is still detectable in punctate bodies within the nucleus of *Top2^f^* mutant cells (Figure S4C). It is known that in flies carrying the null *mod(mdg4)2.2^T16^*, or the hypomorphic *mod(mdg4)2.2^u1^* and *mod(mdg4)2.2^T6^* alleles, the arrangement of Su(Hw) in insulator bodies is greatly disrupted and the protein is randomly distributed throughout the nucleus [14], [38]. Thus, it is possible that the lack of Su(Hw) insulator bodies in *Top2* mutants is due to the inability of Mod(mdg4)2.2 to stably bind to Su(Hw) and bring together distant Su(Hw) insulator sites.

### Topo II does not co-localize with insulator proteins

**Figure 4:**
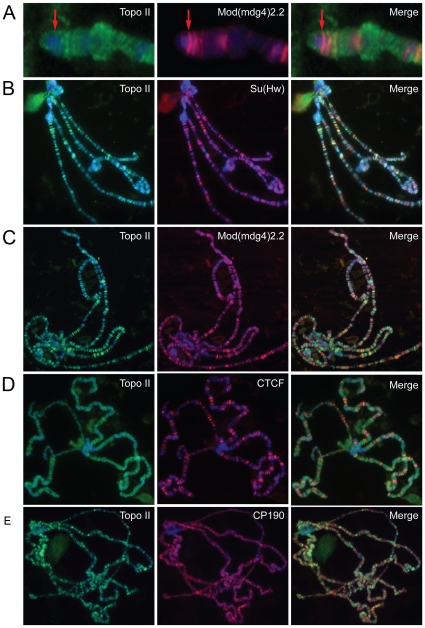
Topo II and insulator proteins do not co-localize on polytene chromosomes. (A) Immunofluorescence staining using α-Topo II and α-Mod(mdg4)2.2 antibodies; the red arrows point to the *yellow* locus indicating that Topo II is present at this location in the *y^2^* allele (red arrow) but Topo II staining is more intense elsewhere in the chromosome. (B) Localization of Topo II and Su(Hw) on polytene chromosomes from wild type larvae. (C) Localization of Topo II and Mod(mdg4)2.2. (D) Localization of Topo II and dCTCF. (E) Localization of Topo II and CP190. The merged images are shown at the right and DAPI is in blue in all panels.

Since Topo II has been found to bind to the *gypsy* retrotransposon insulator *in vitro* [43], it is possible that this protein stabilizes the interaction of Mod(mdg4)2.2 with the insulator complex and that, in its absence, Mod(mdg4)2.2 is unable to bind to other components of the Su(Hw) insulator. To test this possibility, we examined the distribution of Topo II on polytene chromosomes of wild type larvae to determine if this protein could also be found *in vivo* at Su(Hw) insulator sites. To this end we used Topo II antibodies to conduct immunostaining on polytene chromosomes of *y^2^* flies, which have a *gypsy* insertion in the *yellow* locus. In these flies, Topo II is found to localize to *y^2^* as indicated by the co-staining with Mod(mdg4)2.2 (Figure 4A). However, the amount of Topo II at the *y^2^* locus is low compared to other sites in the polytene chromosomes. In addition, Topo II seems to localize primarily at DAPI-stained bands rather than with Mod(mdg4)2.2 or Su(Hw) (Figure 4B and C), suggesting that, although Topo II may be able to bind to *gypsy* retrotransposon sequences, it is not present extensively at other Su(Hw) and Mod(mdg4)2.2 insulator sites in the genome (Figure S5A–B). Topo II also does not co-localize appreciably with dCTCF and CP190 (Figure 4D–E and Figure S5C–D). However, we do see some occurrence of Topo II co-localizing or juxtaposed to insulator bodies in diploid cells (Figure S4C), suggesting a possible transient interaction. Thus, these results suggest that Topo II is not a stable component of the Su(Hw) insulator but may function to modulate insulator activity possibly through a transient interaction.

### Loss of Topo II affects Mod(mdg4)2.2 protein levels

**Figure 5:**
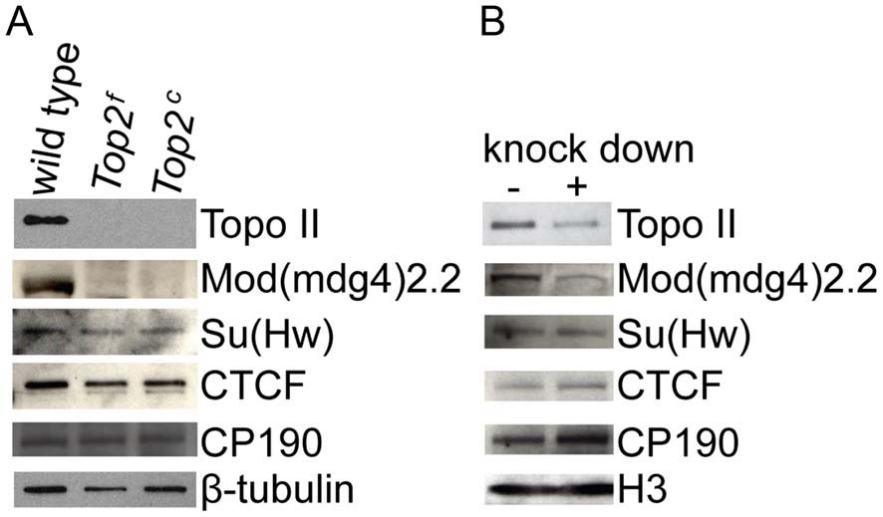
Mod(mdg4)2.2 protein levels are reduced in the absence of Topo II. (A) Western blot analysis of insulator proteins in *Top2^f^* and *Top2^c^* mutants. Protein extracts were prepared from third instar*Top2^f^* and second instar *Top2^c^* larvae. Both Topo II and Mod(mdg4)2.2 protein levels are greatly reduced in the mutants; β-tubulin was used as a loading control. (B) Western blot analysis of dsRNA knockdown of *Top2* in S2 cells; a reduction of both Topo II and Mod(mdg4)2.2 protein levels can be observed with respect to the control sample. H3 was used as a loading control. In both panels Su(Hw), dCTCF and CP190 are not affected by the loss of Topo II.

Since loss of Topo II affects the presence of Mod(mdg4)2.2 at insulator sites and these two proteins do not extensively co-localize on polytene chromosomes, we examined whether the amount of Mod(mdg4)2.2 protein is affected by loss of Topo II. Western blot analysis of *Top2^c^*, *Top2^f^* mutants and *Top2* knockdown in S2 cells were conducted to address this question. Lack of Topo II in *Top2^c^* and *Top2^f^* mutants results in a complete loss of Mod(mdg4)2.2 protein when compared to a wild type sample (Figure 5A), explaining the absence of Mod(mdg4)2.2 on polytene chromosomes of *Top2^f^* mutant larvae. The other *Top2* alleles show little Mod(mdg4)2.2 protein reduction (Figure S6A). In addition, the protein levels of Su(Hw), CP190, and dCTCF are unaffected in *Top2^c^* and *Top2^f^* alleles (Figure 5A). However, prior to the lethal stage of *Top2^c^* and *Top2^f^*, Mod(mdg4)2.2 and TopoII levels can be detected (Figure S6B). Levels of Mod(mdg4)2.2 are also decreased in *Drosophila* cultured cells in which Topo II expression was downregulated using dsRNAs and in *Arm-Gal4;UAS-Top2RNAi* strains (Figure 5B and Figure S6C). The efficiency of Topo II knockdown in these assays ranges between 75%–95% for cultured cells and approximately 85% in the *Arm-Gal4;UAS-Top2RNAi* lines. Consequently, the amount of Mod(mdg4)2.2 is greatly reduced but not completely eliminated in these experiments (Figure 5B and Figure S6C). However, the levels of Su(Hw), CP190, and dCTCF are unchanged in the *Top2* knockdowns. Therefore, the inability of S2 cells lacking Topo II to form Su(Hw) insulator bodies may be due to the loss of Mod(mdg4)2.2 protein. It is possible that in the absence of Topo II there is a decrease in the transcription of the *mod(mdg4)2.2* gene or that the Mod(mdg4)2.2 protein is unable to properly interact with other insulator components and may be directed for degradation.

### Targeted degradation of Mod(mdg4)2.2 in *Top2* mutants

**Figure 6:**
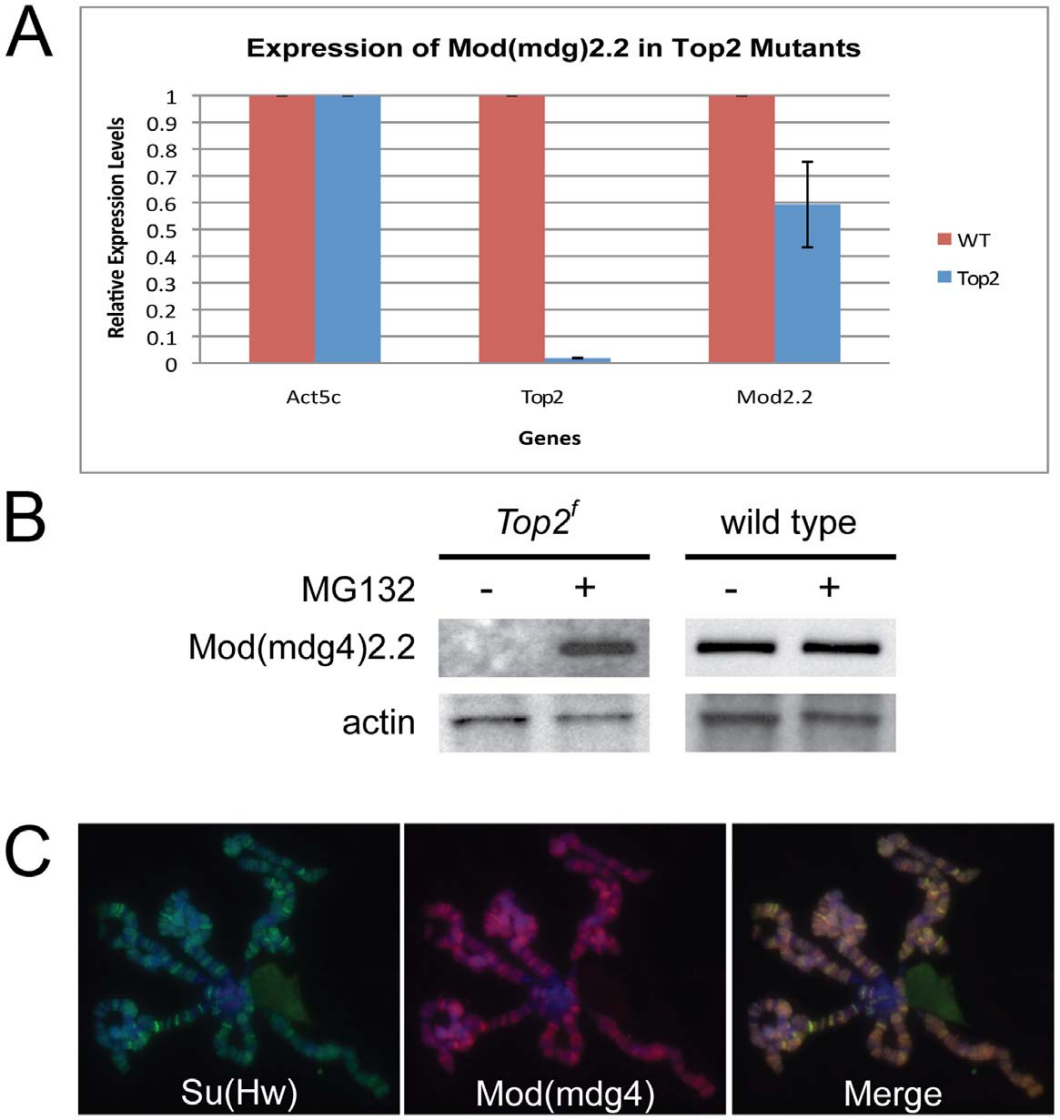
Topoisomerase II modulates Mod(mdg4)2.2 degradation. (A) *Top2* mRNA levels were quantified by qRT-PCR in wild type and mutant *Top2^f^* larvae. *Actin5c* and *RPL32* were used as controls for mRNA levels. *Top2* mRNA is greatly reduced in the *Top2^f^* mutant and *mod(mdg4)2.2* mRNA levels are reduced but only to an average of 60% of wild type. (B) Inhibition of the proteasome by addition of the proteasome inhibitor MG-132 can prevent the degradation of Mod(mdg4)2.2 in the *Top2^f^* mutants. MG-132 has no effect on Mod(mdg4)2.2 protein levels in wild type larvae. (C) Mod(mdg4)2.2 (red) staining is recovered on polytene chromosomes of *Top2^f^* mutant larvae after treatment with the proteasome inhibitor MG-132; Su(Hw) is shown in green and the merge is indicated in yellow.

To distinguish between a possible role for Topo II in the transcription of the *mod(mdg4)2.2* gene, translation of the mRNA, or the stability of the encoded protein, we first analyzed the effects of loss of Topo II on the expression of the *mod(mdg4)2.2* gene. qRT-PCR was used to determine transcript levels of the *Top2* and *mod(mdg4)2.2* genes in wild type and mutant *Top2^f^* larvae. As expected, we see a loss of *Top2* in the *Top2^f^* mutants while *mod(mdg4)2.2* RNA levels are only reduced to an average of 60% of those present in wild type (Figure 6A). This reduction in *mod(mdg4)2.2* transcription only accounts for a fraction of the decrease in Mod(mdg4)2.2 protein observed by western blot analysis and cannot explain the total absence of Mod(mdg4)2.2 protein in *Top2^f^* larvae. Still, it is possible that Topo II affects Mod(mdg4)2.2 protein levels through inhibition of the translation machinery. However, this is less likely due to the fact that a general inhibition of the translation machinery would cause levels of all proteins to diminish, but western analysis only shows a reduction of Mod(mdg4)2.2 while levels of other insulator proteins are unaffected (Figure 5). Furthermore, qRT-PCR analysis using primers to the BTB domain shared by all *mod(mdg4)* isoforms shows a reduction of all *mod(mdg4)* RNAs and not just *mod(mdg4)2.2* (Figure S6D). However, only the Mod(mdg4)2.2 isoform is currently known to interact with the Su(Hw) insulator complex.

To investigate the possible role of Topo II in the degradation of Mod(mdg4)2.2, we inhibited proteasome-dependent degradation using the proteasome inhibitor MG-132, which reduces the degradation of ubiquitin-conjugated proteins. We incubated imaginal discs from mutant *Top2^f^* and wild type third instar larvae with and without the MG-132 proteasome inhibitor for 2 hours. Upon addition of the inhibitor, Mod(mdg4)2.2 protein levels were greatly increased in *Top2^f^* mutants but not in the control treated imaginal discs as visualized by western blot analysis (Figure 6B). Conversely, in the wild type larvae the addition of the inhibitor had little effect on the levels of Mod(mdg4)2.2 when compared to the control treated imaginal discs (Figure 6B). This suggests that the Mod(mdg4)2.2 protein is more susceptible, and possibly actively targeted for degradation, in cells lacking Topo II. To determine the fate of the Mod(mdg4)2.2 protein that accumulates after inhibition of proteasome function, salivary glands from *Top2^f^* mutant larvae were incubated with MG-132. As in the case of the imaginal disc cells, this treatment results in increased Mod(mdg4)2.2 accumulation and the protein appears to properly localize to polytene chromosomes and co-localizes with Su(Hw) (Figure 6C). Thus, Topo II seems to be required to prevent the degradation of Mod(mdg4)2.2 and to allow for proper organization of the Su(Hw)/Mod(mdg4)2.2 insulator complex.

### Topoisomerase II directly interacts with Mod(mdg4)2.2

**Figure 7:**
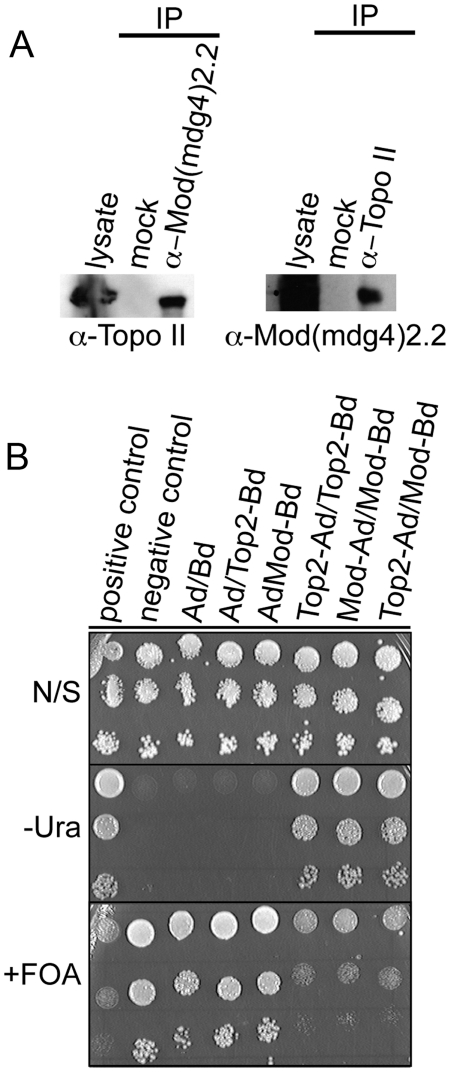
Mod(mdg4)2.2 and Topo II directly interact. (A) Topo II and Mod(mdg4)2.2 co-immunoprecipitate. The left panel shows extracts treated with antibodies to Mod(mdg4)2.2 and subjected to western analysis using α-Topo II antibodies. The right panel shows extracts treated with antibodies to Topo II and subjected to western analysis using α-Mod(mdg4)2.2 antibodies. (B) Assay for direct interaction between Topo II and Mod(mdg4)2.2. Yeast expressing Topo II and Mod(mdg4)2.2 fused to either the GAL4 activation domain (AD) or DNA binding domain (BD) were spotted onto plates with nonselective (N/S), lacking uracil (−URA), or medium containing 5FOA (+FOA). Interaction is indicated by growth on −URA and the lack of growth on the FOA plates.

To investigate whether the effect of Topo II on Mod(mdg4)2.2 stability is direct or indirect we used co-immunoprecipitation (co-IP) and yeast two-hybrid experiments to determine whether the two proteins interact. Results from the co-IP experiments show that Topo II and Mod(mdg4)2.2 immunoprecipitate one another (Figure 7A). In support of this conclusion, yeast two-hybrid analyses indicate that Topo II and Mod(mdg4)2.2 can directly interact (Figure 7B). The positive controls, Topo II-Ad (activation domain)/Topo II-Bd (binding domain) and Mod(mdg4)2.2-Ad/Mod(mdg4)2.2-Bd, show interaction-dependent phenotypes similar to Topo II-Ad/Mod(mdg4)2.2-Bd while the negative controls do not. These results, combined with the immuno-colocalization studies, suggest that Topo II and Mod(mdg4)2.2 do interact but this interaction may be transient and most likely does not take place on the chromatin. Thus, it appears that the effect of Topo II on the Su(Hw) insulator may be mediated by a direct association between Mod(mdg4)2.2 and Topo II that ultimately leads to modulation of Mod(mdg4)2.2 levels within the cell.

## Discussion

Insulators mediate intra- and inter- chromosomal interactions and in doing so they organize the chromatin fiber within the eukaryotic nucleus [10]. Recent evidence suggests that this organization is cell type-specific [17], [21], [44], [45], [46] and that it plays a role in both the control of gene expression [47], [48] and in the epigenetic inheritance of imprinted expression patterns [49]. Topo II is an essential component of a variety of nuclear processes. Reports suggesting the presence of this protein bound to sequences of the insulator present in the *gypsy* retrotransposon [43] prompted us to examine the possibility of a role for Topo II in insulator function. In support of this possibility, we find that mutations in the *Top2* gene interfere with the function of the insulator present in the *gypsy* retrotransposon. This is confirmed by the presence of Topo II at the *yellow* locus of *y^2^* flies carrying an insertion of the *gypsy* retrotransposon in the *yellow* gene. However, Topo II is absent from both Su(Hw) and dCTCF endogenous insulator sites throughout the *Drosophila* genome indicating that Topo II is not a stable component of these insulator complexes. Nevertheless, downregulation of the Topo II protein using RNAi results in disruption of insulator bodies formed by the Su(Hw) protein. Furthermore, mutation of Topo II affects the binding of Mod(mdg4)2.2 but not of other insulator proteins to polytene chromosomes, suggesting that Topo II has a role in regulating Su(Hw) insulator activity by facilitating Mod(mdg4)2.2 interaction with DNA. These results are significant because they highlight a potential regulatory pathway by which Mod(mdg4)2.2 can modulate the activity of Su(Hw) insulators. Previous work has revealed common mechanisms used by different insulators in *Drosophila* such as the general requirement for the protein CP190. We have recently shown that three previously characterized *Drosophila* insulators thought to be unrelated, *gypsy*, *Fab8* and *scs'*, actually share the CP190 protein and perhaps contain different isoforms of Mod(mdg4) [25], [50]. Based on their distribution with respect to gene features, we have suggested that these different insulators may play distinct roles in gene expression. The fact that loss of Topo II only affects the Su(Hw) insulator subclass supports the conclusion of a distinct and specific role for this insulator. It is possible that dCTCF and BEAF insulators contain other Mod(mdg4) isoforms that are also affected by TopoII or they may be regulated by distinct mechanisms yet to be identified.

It has been proposed that insulators form loops through the interaction of individual insulator sites coming together at specific nuclear locations named insulator bodies. This model is in part supported by recent 3C analyses of intra-chromosomal interactions suggesting that several insulator sites can interact (A. Bushey, K. Van Bortle and V. Corces unpublished data). In *Drosophila* cells, these insulator bodies are thought to contain both sites of the *gypsy* retrotransposon as well as endogenous insulators. Thus, their stability would depend on proteins associated with endogenous as well as *gypsy* retrotransposon sites. We therefore examined the appearance of these insulator bodies as a way to determine how disruption of Topo II may impact the functional state of endogenous insulators along with general chromatin organization. Surprisingly, downregulation of the Topo II protein using RNAi results in disruption of insulator bodies formed by the Su(Hw) protein, suggesting that Mod(mdg4)2.2 is important for the nucleation of insulator bodies. Since mutation of Topo II affects the binding of Mod(mdg4)2.2 but not of other insulator proteins to polytene chromosomes, the results suggest that interaction of Mod(mdg4)2.2 with chromatin is necessary for the formation of insulator bodies.

Providing further insight into the relationship between Topo II and Mod(mdg4)2.2, co-immunoprecipitation and yeast two hybrid experiments suggest that the effect of Topo II on Su(Hw) insulator function is mediated by a direct interaction between Mod(mdg4)2.2 and Topo II. Interestingly, the association between the two proteins cannot be visualized on polytene chromosomes, suggesting that the interaction is transient or that it occurs in the nucleoplasm but not directly on the DNA. The lack of Mod(mdg4)2.2 protein on chromosomes is partly due to its downregulation at the level of RNA synthesis but, more dramatically, at the level of protein degradation, suggesting that the interaction between Topo II and Mod(mdg4)2.2 may bring the latter into contact with proteins that protect it from proteasome targeting or result in the modification of the Mod(mdg4)2.2 protein preventing its degradation. This ability of Topo II to protect Mod(mdg4)2.2 protein from proteasome-dependent degradation is similar to that observed in other systems [51], [52]. For example, when Topo II function is inhibited by the Topo II poison R16 in human cells, a reduction of the DNA damage check point protein Chk1 is observed without greatly effecting Chk1 mRNA levels [52]. It is believed that Chk1 reduction contributes to the anticancer effects of R16 thus leading to apoptotic induction and cell death. It is also possible that the targeted reduction of Mod(mdg4)2.2 could play a function in cell death, since a splice variant of Mod(mdg4), Doom, has a function in apoptosis [53]. Thus, it is conceivable that loss of Topo II triggers the cell to turn on the proteasome pathway to target a subset of proteins involved in apoptosis or cell survival.

## Materials and Methods

### Genetics, *Drosophila* strains and RNAi knockdown in flies

All stocks were cultured under standard conditions on yeast-agar medium at 25°C. The *UAS-Top2RNAi* (transformant ID 30625) and the *UAS-Su(Hw)RNAi* (transformant ID 10724) flies were obtained from the Vienna *Drosophila* RNAi Center and crossed to the different *Gal4* driver lines. The P-element insertions into the *Top2* gene, *Top2^c05388^*, *Top2^d05357^*, *Top2^f05145^*, *Top2^LA00892^* and *Top2^MB04073^*, were obtained from the Bloomington *Drosophila* Stock Center at Indiana University. The following strains were used for enhancer blocking assays, RNAi knockdowns and genetic analysis: *Actin5c-Gal4*, *Ptc-Gal4*, *ey-Gal4*, *GMR-Gal4*, *CyO, Actin5c-GFP*, *Arm-Gal4*, *C96-Gal4* (a gift from B. Yedvobnick), *Df(2L)Exel9043*, and *y^2^ w ct^6^*. The time and level of expression of the *Actin5c-Gal4* and *Arm-Gal4* were reported by Ahmad and Henikoff [35].

### Topoisomerase II RNAi knockdown in cell culture

RNAi knockdown in cultured *Drosophila* S2 cells was conducted as per the *Drosophila* RNAi Screening Center (DRSC) protocol [40]. Primers TopoIIA5′ TAATACGACTCACTATAGGGTTTGCCAGAGCGATATCTC, TopoIIA3′ TAATACGACTCACTATAGGGCCATAGTGGCTCGATCTTTT, TopoIIB5′ TAATACGACTCACTATAGGGCACAGCGACAGAAGCATCAT, TopoIIB3′ TAATACGACTCACTATAGGGTTCTTGTATTCCCTCGTGGC, LacZ5′ TAATACGACTCACTATAGGGGGTTTCCGCGAGGT, and LacZ3′ TAATACGACTCACTATAGGGGTCGCACAGCGTGTAC where used to make amplicons targeting the second and fourth exons of *Top2*, and the *LacZ* gene. Both second and fourth exon amplicons of *Top2* have no off target sites; the *LacZ* amplicon does not target any *Drosophila* gene. Cells were incubated with dsRNAs for 3 days prior to fixation and western analysis.

### Immunofluorescence analysis of diploid cells and polytene chromosomes

S2 cells were fixed with 3.7% formaldehyde in a solution of 0.1% Triton X-100 and 0.1 M sodium phosphate buffer pH 7.2 (NaP/TX-buffer) for 30 min and blocked for 30 min in NaP/TX-buffer containing 5% normal goat serum. Cells were then attached to 0.01% poly-L-lysine treated slides for 10 min. The liquid was aspirated and cells were incubated overnight at 4°C in a humidified chamber containing primary antibodies at 1∶1000 dilution for α-dCTCF, 1∶2000 α-CP190, 1∶1000 α-Su(Hw), 1∶1000 α-Mod(mdg4)2.2, 1∶3000 for α-Topo II and 1∶300 α-Dlg. The cells were washed 3 times for 10 min with NaP/TX-buffer and incubated with Alexa Fluor 488- and/or 594-conjugated goat anti-rabbit, -guinea pig, -rat, or -mouse IgG in NaP/TX/NGS in a 1∶1000 dilution 2 hr at room temperature. The cells were washed twice with NaP/TX-buffer, once with NaP-buffer and incubated for 10 min with DAPI [0.5 µg/ml]. Cells were then rinsed once with NaP buffer and mounted with Vectashield antifade mounting medium.

Immunostaining of polytene chromosomes was carried out as described [37] except that Alexa Fluor secondary antibodies and the following primary antibodies where used: α-dCTCF (1∶100), α-JIL-1 (1∶100), α-Mod(mdg4)2.2 (1∶200), α-Su(Hw) (1∶100), α-CP190 (1∶300), α-Topo II (1∶200).

### Quantitative real-time PCR analysis

RNA was isolated from wild type and *Top2* larvae using the QiagenRNeasy kit (catalog #74104) with on-column DNA digestion (catalog #79254) and cDNA synthesis was performed using the Applied Biosystems High Capacity cDNA Reverse Transcription Kit (catalog #4368814). Real-time PCR analysis was then used to quantify levels of *Act5C*, *RPL32*, *Top2*, *mod(mdg4)2.2*, and all *mod(mdg4)*RNAs. Primers: Act5C 5′-GTCGTCTAATCCAGAGACAC, 3′-CCAGAGCAGCAACTTCTTCG; RPL32 5′-CCGCTTCAAGGGACAGTATC, 3′-GACAATCTCCTTGCGCTTCT; Top25′-GCGAAGCTCTGCAACATATTC, 3′-GAAGTCCTTGATCTGCACATC; Mod(mdg4)2.2 5′-CACGAAGGGCGGTGTCAAGC, 3′-CACGTGCTCGCCCTCGTAAG; Mod(mdg4)BTB 5′-GATCGTTATCCGTTAGCCCC, 3′-CACCCACGCTATCGTATTCC. Expression levels were normalized to Act5C and RPL32 with no significant difference; thus, only Act5c is shown.

### Western blot and immunoprecipitation analyses

For western analysis, extracts from S2 cells and imaginal tissue from third instar larvae were prepared using standard protocols and run on tris-glycine gels using SDS sample buffer [37]. Co-immunoprecipitation was conducted as described [15]. The Millipore SNAP i.d. protein detection system was used for all the protein immunodetections following the manufacturer's protocol. Blots were probed with the following primary antibodies α-CP190 (1∶6000), α-dCTCF (1∶3000), α-Su(Hw) (1∶3000), α-Topo II (1∶10000), α-β-tubulin (1∶1500), α-H3 (1∶10000), α-Mod(mdg4)2.2 (1∶3000) and appropriate HRP-conjugated secondary antibodies (1∶3000). The signal was detected using Thermo Scientific chemiluminescent substrates following the manufacturer's protocol.

### Proteasome inhibition assay

Salivary glands and imaginal discs from wild type and *Top2^f^* third instar larvae were dissected and placed into serum free HyClone CCM-3 Insect medium. The salivary glands were then transferred to HyClone medium containing either 50 µM MG-132 proteasome inhibitor dissolved in DMSO or HyClone with DMSO only and incubated for 2 h at 25°C. Western and immunofluorescence analyses were then conducted as described above.

### Yeast two-hybrid assay

The yeast two-hybrid assay was conducted using the Invitrogen ProQuest Two-Hybrid System according to the manufacture's protocol. Full length *Top2* and *mod(mdg4)2.2* cDNAs were PCR cloned into the Gateway pENTR vector and shuttled to pDEST22-AD (activation domain) or pDEST32-BD (binding domain) vectors. The Topo II cDNA was a gift of Dr. T. Hsieh. Controls provided by the kit are negative for mutants mt1-RalGDS-BD/Krev1AD, mt2-RalGDS/Krev1-AD and positive for wt-RalGDS-BD/Krev1-AD. In addition, the empty activation domain/DNA binding domain (Ad/Bd), empty Ad/Top2-Bd and empty Ad/Mod(mdg4)2.2-Bd plasmids where used as negative controls.

## Supporting Information

Figure S1 Downregulation of *Top2* in *Drosophila* using tissue specific Gal4 drivers. (A) Quantification of *Top2* transcript using qRT-PCR in animals in which *Top2* expression was downregulated using RNAi under the control of *Arm-Gal4*. Significant reduction of *Top2* can be observed compared to wild type. (B) *Top2* RNAi under the control of the tissue specific Gal4 driver *C96-Gal4* shows reduction of Topo II at the dorsal-ventral boundary (red arrow) as visualized in wing discs by immunofluorescence microscopy. Topo II is in green and *Drosophila* discs large (Dlg), a marker for wing margin cells, is in red.
(TIF)

Figure S2 Structure of the *Top2* locus and western analysis of dsRNA knockdowns. (A) A schematic diagram of the *Top2* locus, detailing the location of the *Top2* mRNA, the *Top2* RNAi amplicons (DRSC03459, DRSC36057) used for dsRNA knockdowns and P-element insertions in intron 2 of the *Top2* gene. (B) Western blot analysis of Topo II after a 72 hr incubation of S2 cells with dsRNA made using either exon 2 or exon 4 amplicons. β-tubulin is used as a loading control.
(TIF)

Figure S3 Characterization of *Top2* alleles. (A) Quantification of *Top2* transcript levels in each P-element-induced allele, *Top2^LA^*, *Top2^d^*, *Top2^MB^*, *Top2^f^*, *Top2^c^* and wild type. (B) Western blot analysis of Topo II levels in wild type, *Top2^LA^*, *Top2^d^*, *Top2^MB^*, *Top2^f^* and *Top2^c^* fly lines.
(TIF)

Figure S4 Immunofluorescence microscopy of polytene chromosomes from*Top2^f^* mutants and *Drosophila* cultured cells. (A) Staining of polytene chromosomes from wild type and *Top2^f^* flies with antibodies against Topo II (green) and dCTCF (red). dCTCF is unaffected in *Top2^f^* whereas TopoII is absent. (B) Staining of polytene chromosomes from wild type and *Top2^f^* flies with antibodies against JIL-1 (green) and Mod(mdg4)2.2 (red). Mod(mdg4)2.2 is absent in polytene chromosomes from larvae lacking Topo II. (C) The Mod(mdg4)2.2 (red) foci at insulator bodies are absent in *Top2^f^* mutant animal tissue. Topo II is labeled in green.
(TIF)

Figure S5 Localization of Topo II with respect to insulator proteins. (A–D) Magnified regions of polytene chromosomes from Figure 4. Yellow arrows indicate co-localization of Topo II and insulator proteins. (E) Nuclear localization of Topo II and Mod(mdg4)2.2 in S2 cells. In all panels Topo II is green and the corresponding insulator protein is labeled in red.
(TIF)

Figure S6 Insulator protein levels in *Top2* alleles. (A) Western blots of Topo II, Mod(mdg4)2.2, and Su(Hw) using protein extracts from larval imaginal tissue of *Top2* alleles. (B) Western analysis of *Top2^f^* and *Top2^c^* alleles prior to their lethal stage. *Top2^c^* larvae were collected early in 2^nd^ instar and *Top2^f^* larvae were collected by mid 2^nd^ instar. Mod(mdg4)2.2 and Topo II are still detectable at these stages of development. (C) Western analysis of Topo II, Mod(mdg4)2.2 and Su(Hw) in *Arm-Gal4;UAS-Top2RNAi* larvae knockdowns. (D) *mod(mdg4)* mRNA levels were quantified by qRT-PCR in wild type and mutant *Top2^f^* larvae using primers for the BTB domain shared by all isoforms; total *mod(mdg4)* transcript levels are reduced.
(TIF)
