# Supplementary figures and images for: DNA Topoisomerase II Modulates Insulator Function in Drosophila
Source: PLoS One. 2011 Jan 27;6(1):e16562. doi: 10.1371/journal.pone.0016562 (PMC3029388; doi:10.1371/journal.pone.0016562)

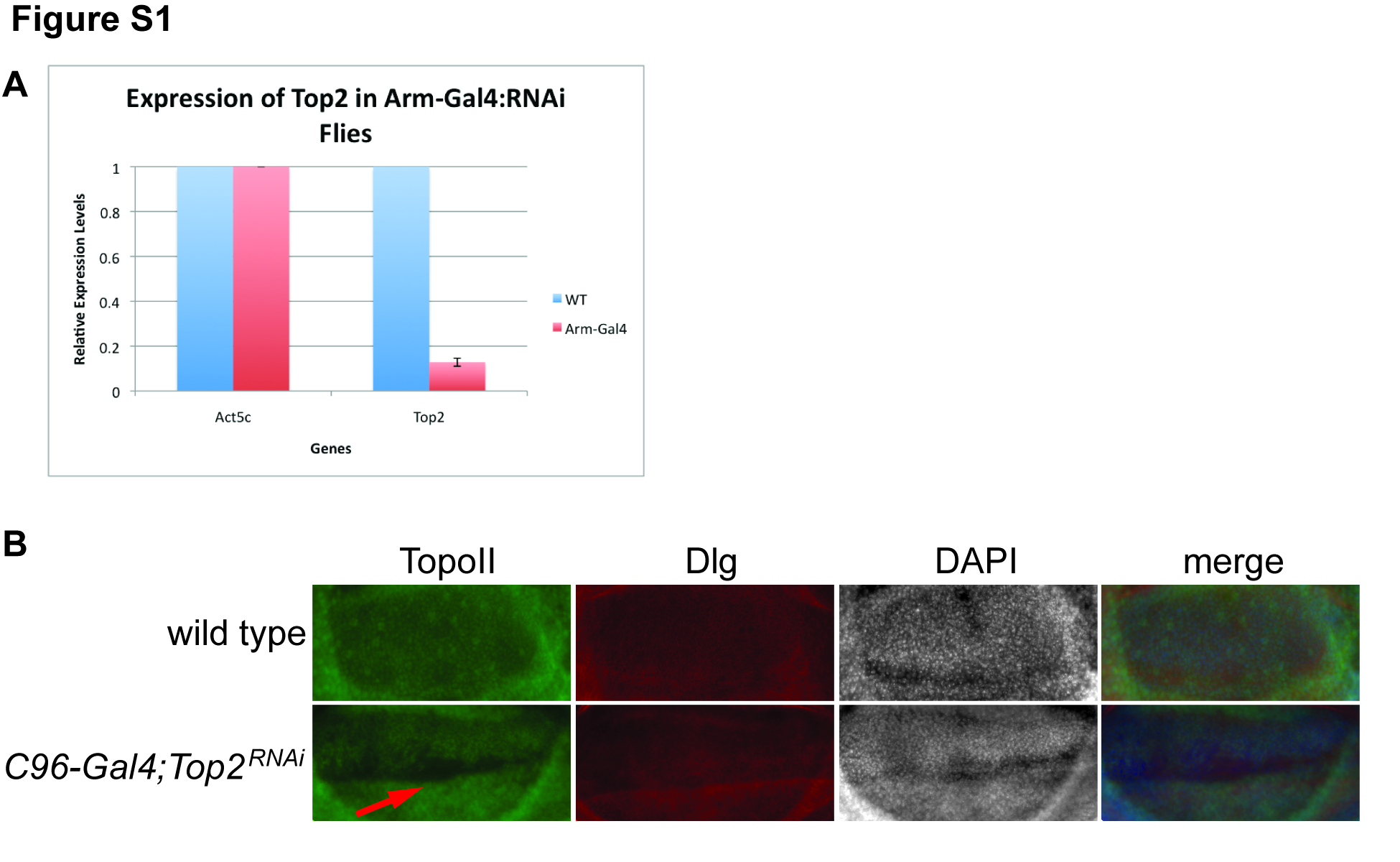

Supplement: Figure S1 — Downregulation of Top2 in Drosophila using tissue specific Gal4 drivers. (A) Quantification of Top2 transcript using qRT-PCR in animals in which Top2 expression was downregulated using RNAi under the control of Arm-Gal4. Significant reduction of Top2 can be observed compared to wild type. (B) Top2 RNAi under the control of the tissue specific Gal4 driver C96-Gal4 shows reduction of Topo II at the dorsal-ventral boundary (red arrow) as visualized in wing discs by immunofluorescence microscopy. Topo II is in green and Drosophila discs large (Dlg), a marker for wing margin cells, is in red. (TIF) [file pone.0016562.s001.tif]

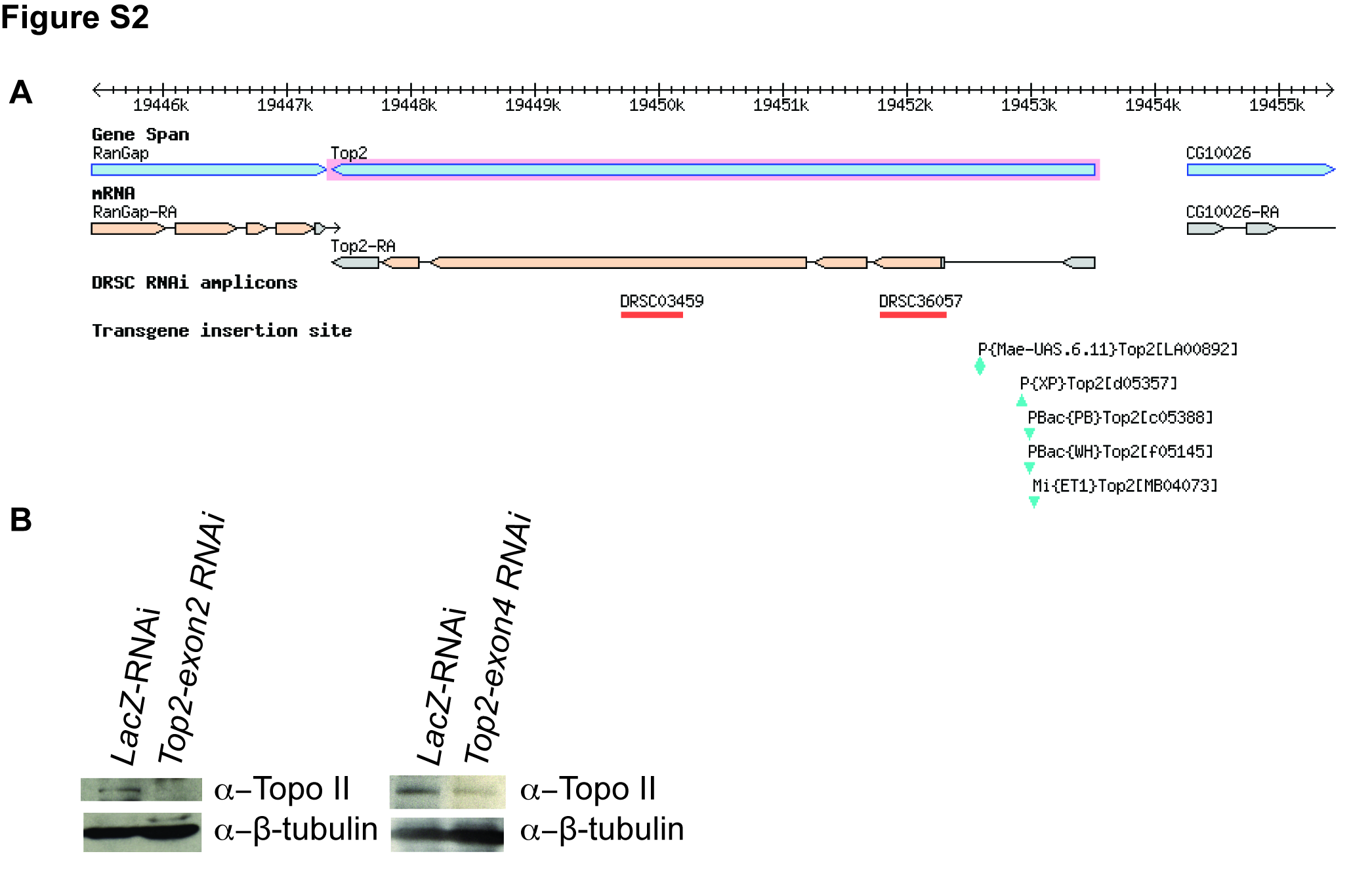

Supplement: Figure S2 — Structure of the Top2 locus and western analysis of dsRNA knockdowns. (A) A schematic diagram of the Top2 locus, detailing the location of the Top2 mRNA, the Top2 RNAi amplicons (DRSC03459, DRSC36057) used for dsRNA knockdowns and P-element insertions in intron 2 of the Top2 gene. (B) Western blot analysis of Topo II after a 72 hr incubation of S2 cells with dsRNA made using either exon 2 or exon 4 amplicons. β-tubulin is used as a loading control. (TIF) [file pone.0016562.s002.tif]

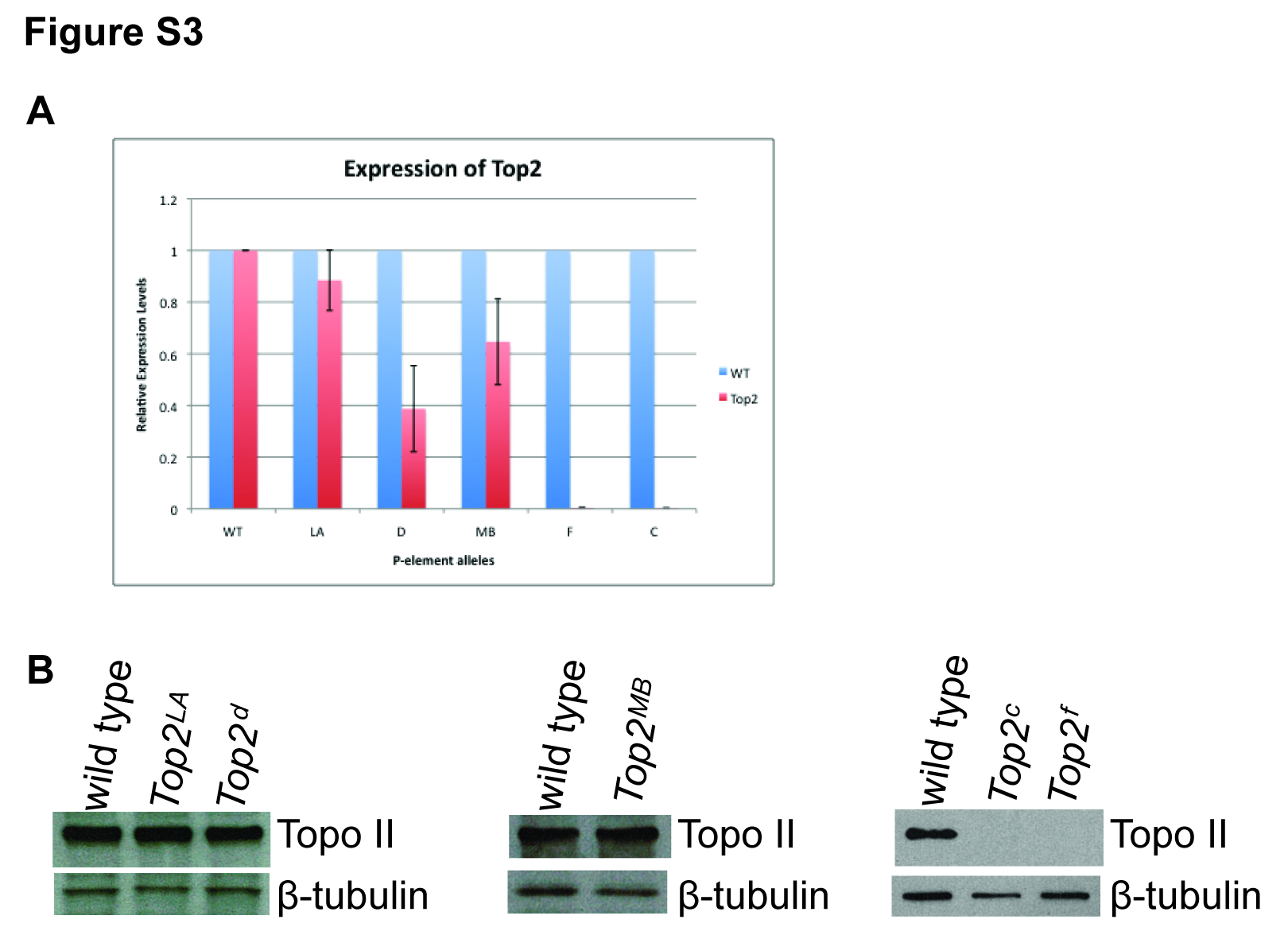

Supplement: Figure S3 — Characterization of Top2 alleles. (A) Quantification of Top2 transcript levels in each P-element-induced allele, Top2LA, Top2d, Top2MB, Top2f, Top2c and wild type. (B) Western blot analysis of Topo II levels in wild type, Top2LA, Top2d, Top2MB, Top2f and Top2c fly lines. (TIF) [file pone.0016562.s003.tif]

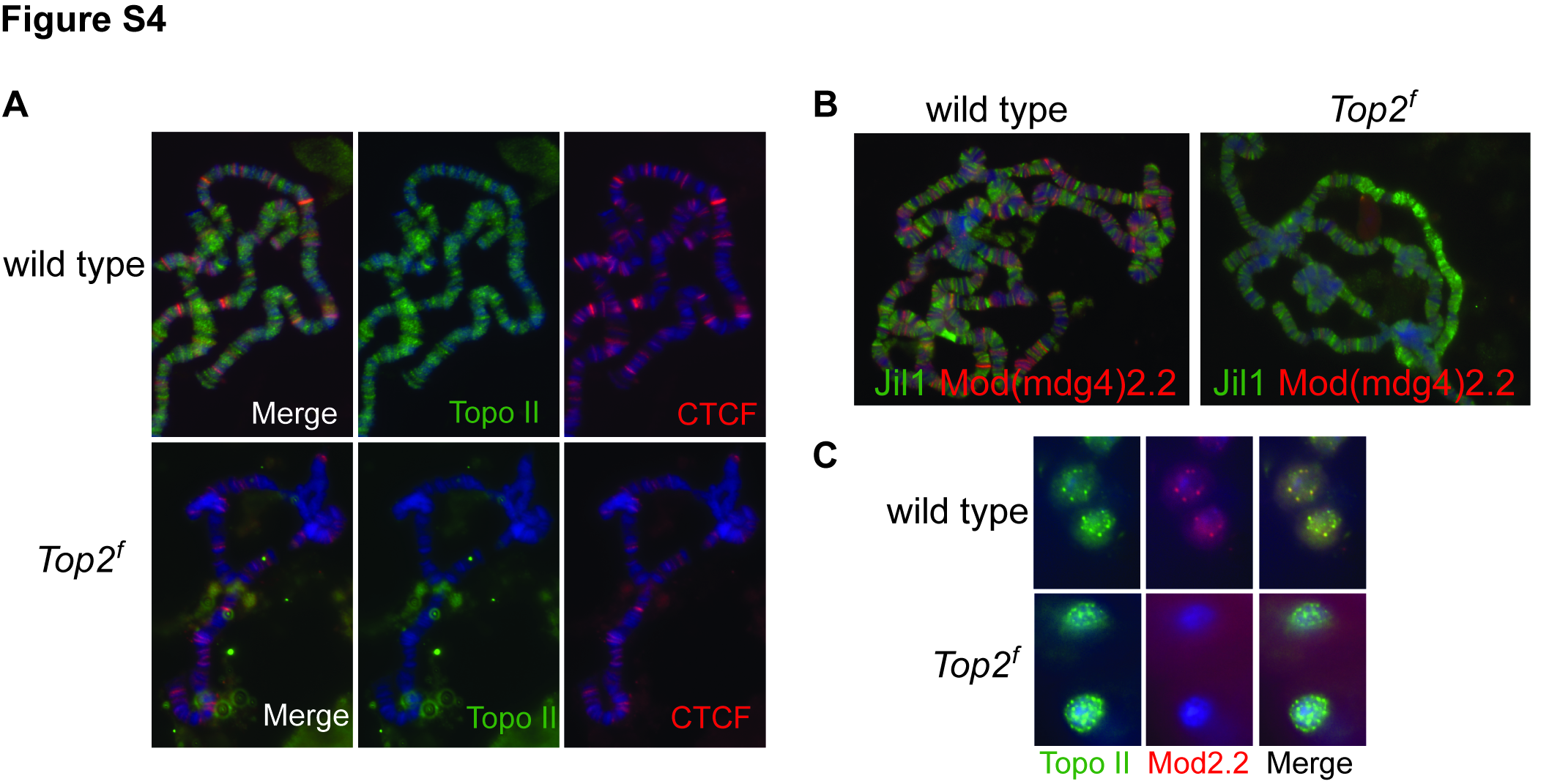

Supplement: Figure S4 — Immunofluorescence microscopy of polytene chromosomes fromTop2f mutants and Drosophila cultured cells. (A) Staining of polytene chromosomes from wild type and Top2f flies with antibodies against Topo II (green) and dCTCF (red). dCTCF is unaffected in Top2f whereas TopoII is absent. (B) Staining of polytene chromosomes from wild type and Top2f flies with antibodies against JIL-1 (green) and Mod(mdg4)2.2 (red). Mod(mdg4)2.2 is absent in polytene chromosomes from larvae lacking Topo II. (C) The Mod(mdg4)2.2 (red) foci at insulator bodies are absent in Top2f mutant animal tissue. Topo II is labeled in green. (TIF) [file pone.0016562.s004.tif]

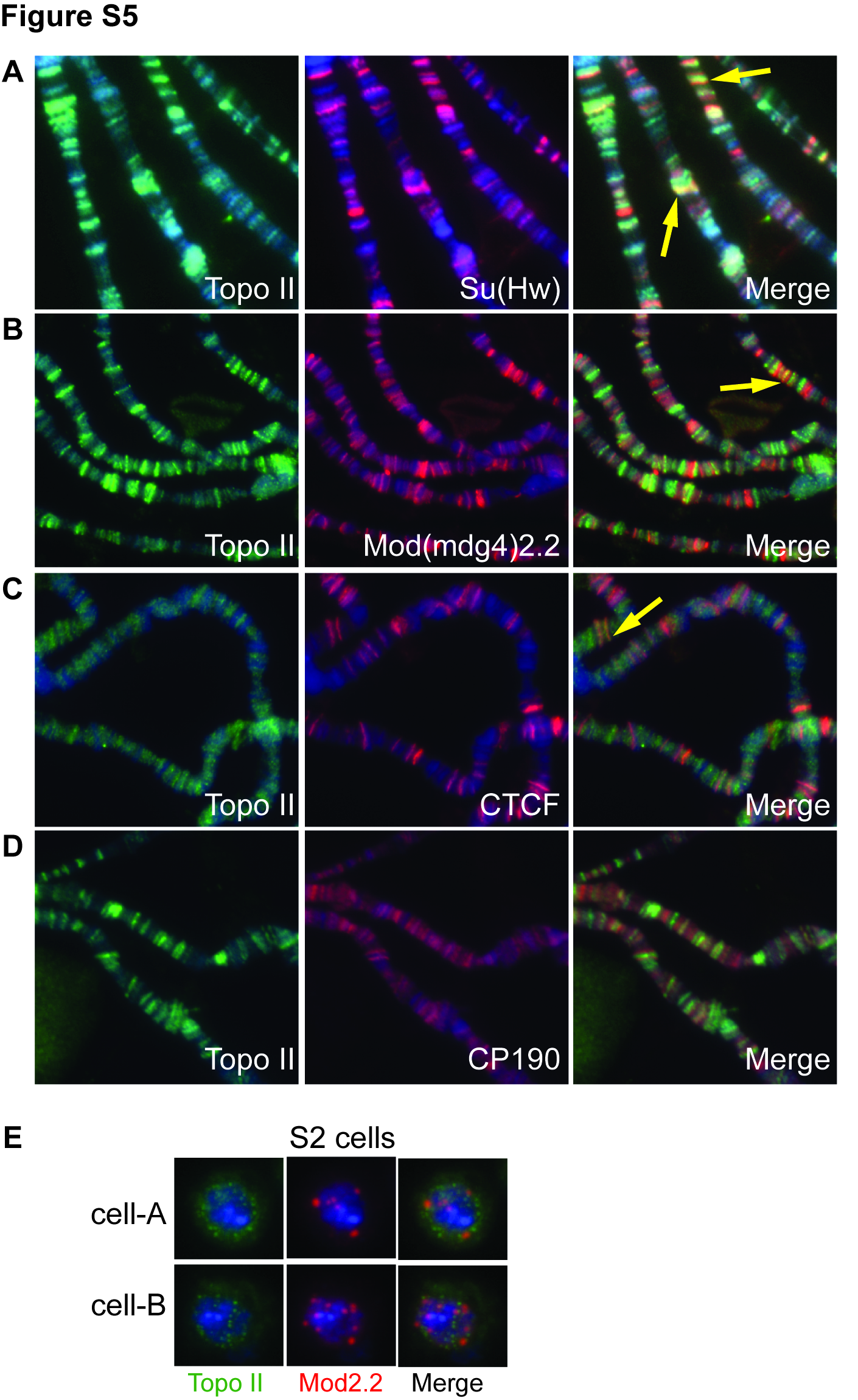

Supplement: Figure S5 — Localization of Topo II with respect to insulator proteins. (A–D) Magnified regions of polytene chromosomes from Figure 4. Yellow arrows indicate co-localization of Topo II and insulator proteins. (E) Nuclear localization of Topo II and Mod(mdg4)2.2 in S2 cells. In all panels Topo II is green and the corresponding insulator protein is labeled in red. (TIF) [file pone.0016562.s005.tif]

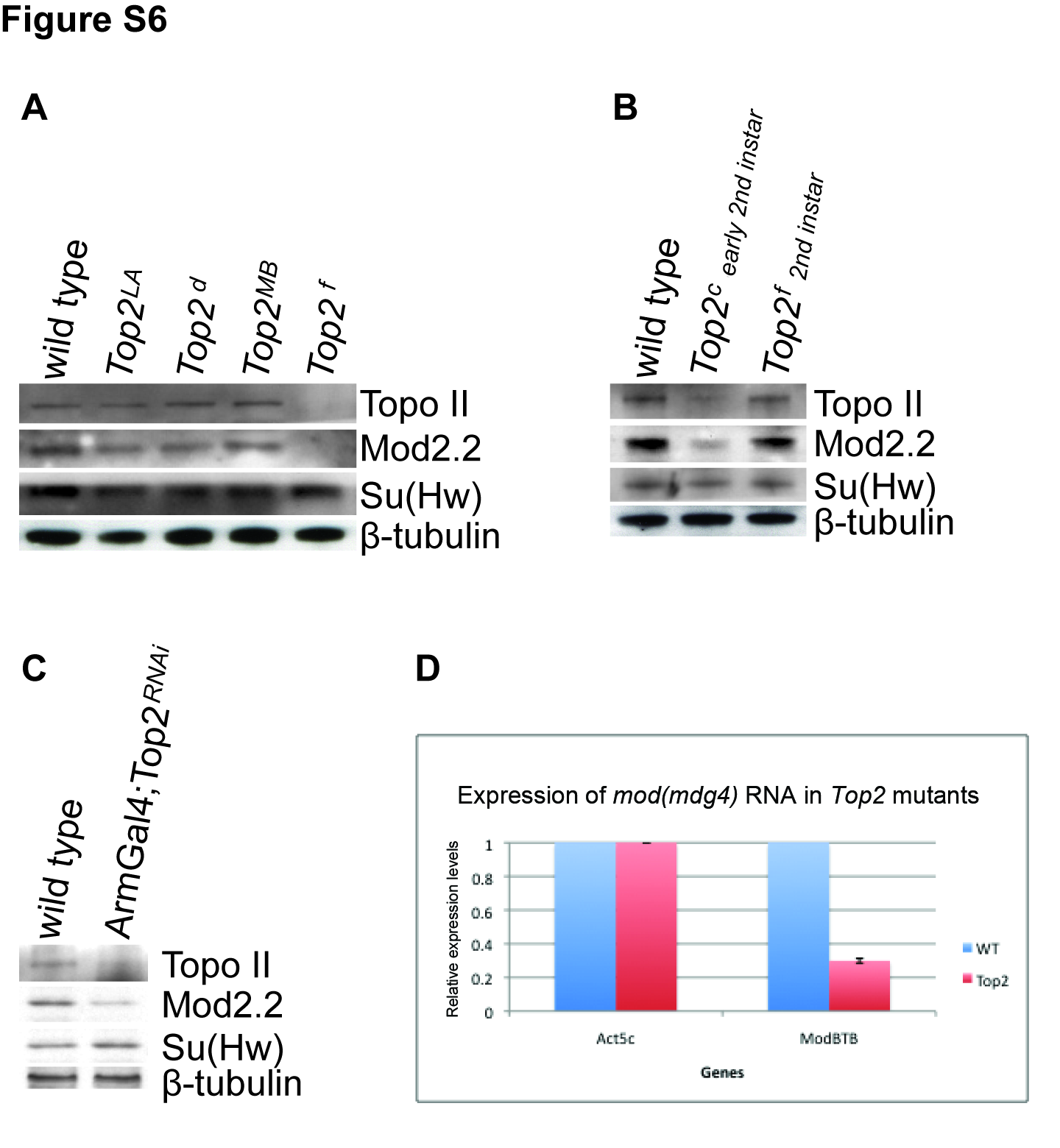

Supplement: Figure S6 — Insulator protein levels in Top2 alleles. (A) Western blots of Topo II, Mod(mdg4)2.2, and Su(Hw) using protein extracts from larval imaginal tissue of Top2 alleles. (B) Western analysis of Top2f and Top2c alleles prior to their lethal stage. Top2c larvae were collected early in 2nd instar and Top2f larvae were collected by mid 2nd instar. Mod(mdg4)2.2 and Topo II are still detectable at these stages of development. (C) Western analysis of Topo II, Mod(mdg4)2.2 and Su(Hw) in Arm-Gal4;UAS-Top2RNAi larvae knockdowns. (D) mod(mdg4) mRNA levels were quantified by qRT-PCR in wild type and mutant Top2f larvae using primers for the BTB domain shared by all isoforms; total mod(mdg4) transcript levels are reduced. (TIF) [file pone.0016562.s006.tif]
